# Redirection of Human Cancer Cells upon the Interaction with the Regenerating Mouse Mammary Gland Microenvironment

**DOI:** 10.3390/cells2010043

**Published:** 2013-01-10

**Authors:** Sonia M. Rosenfield, Gilbert H. Smith

**Affiliations:** Cell and Cancer Biology Branch (CCBB), Center for Cancer Research, National Cancer Institute, NIH, Building 37, Room 1112B, 37 Convent Drive Bethesda, MD 20892, USA; E-Mail: sonia.rosenfield@nih.gov

**Keywords:** human cancer cells, redirecting cell fate, progenitor cell, mammary gland, microenvironment

## Abstract

Tumorigenesis is often described as a result of accumulated mutations that lead to growth advantage and clonal expansion of mutated cells. There is evidence in the literature that cancer cells are influenced by the microenvironment. Our previous studies demonstrated that the mouse mammary gland is capable of redirecting mouse cells of non-mammary origins as well as Mouse Mammary Tumor Virus (MMTV)-neu transformed cells toward normal mammary epithelial cell fate during gland regeneration. Interestingly, the malignant phenotype of MMTV-neu transformed cells was suppressed during serial transplantation experiments. Here, we discuss our studies that demonstrated the potential of the regenerating mouse mammary gland to redirect cancer cells of different species into a functional tumor-free mammary epithelial cell progeny. Immunochemistry for human specific CD133, mitochondria, cytokeratins as well as milk proteins and FISH for human specific probe identified human epithelial cell progeny in ducts, lobules, and secretory acini. Fluorescent In Situ Hybridization (FISH) for human centromeric DNA and FACS analysis of propidium iodine staining excluded the possibility of mouse-human cell fusion. To our knowledge this is the first evidence that human cancer cells of embryonic or somatic origins respond to developmental signals generated by the mouse mammary gland microenvironment during gland regeneration *in vivo*.

## 1. Introduction

Tumorigenesis has been previously described as a complex genetic disease where accumulated mutations and epigenetic changes in a cell can cause the activation of oncogenes, inactivation of tumor suppression genes and deregulation of stability genes [1,2]. Accumulated mutations result in deregulated signaling pathways that favor the proliferation of the mutated cells and thus tumor formation [2]. However, there is evidence in the literature that the normal microenvironment of cancer cells has the potential to suppress tumorigenesis and that this is possibly achieved by regulating the cancer cells position within their niche and the signaling with surrounding cells [1]. 

Dean Felsher previously discussed a model where tumorigenesis is the result of the accumulation of genetic alterations that occur only if the mutated cells are in the context of a tumor-permissive microenvironment [1]. Studies done using a MYC conditional osteosarcoma transgenic mouse model supported this proposed model and gave evidence that oncogene-driven-malignant transformation can be suppressed if the cancer cells are in a tumor non-permissive location within the niche [3]. As expected, the transient inactivation of the MYC oncogene in the presence of doxycycline treatments caused tumors in mice to regress [3]. Surprisingly, the removal of doxycycline and thus reactivation of the MYC oncogene did not result in tumors regrowth [3]. It was found that the reactivation of the MYC oncogene led to apoptosis of the tumor cells [3]. Furthermore, transplantation experiments of enucleated oocytes that were injected with nuclei of tumor cells demonstrated that tumor cells could be regulated by the ‘normal’, non-tumorigenic microenvironment of the enucleated oocyte [4]. Even though the resulting chimeric mice were predisposed towards tumor formation, the majority of their tissues were normal [4]. 

Further studies have confirmed that tumorigenesis is suppressed only if the cancer cells are located in a tumor non-permissive microenvironment such as during tissue development [5,6]. The Rous sarcoma virus causes aggressive tumors when it is injected directly into the wings of chickens [7,8]. Experiments where cultured chicken embryonic fibroblasts were infected with tagged pp60^src^, the non-receptor protein tyrosine kinase that mediates the Rous sarcoma virus’s activity, demonstrated that the infected cells acquired foci formation potential and anchorage independent growth, typical characteristics of transformed cells [6]. The oncogenic potential of the tagged pp60^src^ virus was further confirmed *in vivo* by intramuscular injections of the virus in the wings of hatchling chickens [6]. Fast growing tumors formed in almost 100% of the injected chickens [6]. However, when the tagged pp60^src^ virus was used to infect chicken limb embryo cells *in ovo*, no tumors formed and the virus was detected in the majority of cell types in the infected embryos [6,9]. Overall, these findings demonstrate that tumorigenesis is not exclusively dependent on genetic and epigenetic changes that occur within the cancer cells but also on the signals generated in the surrounding normally developing tissues. 

The concept that cancer cells can respond to developmental stimuli and differentiate contributing to normal tissues development has been explored since the late 1950s and early 1960s [5,6,10,11,12,13,14,15,16,17]. In 1974, Brinster showed that embryonal carcinoma cells gave rise to chimeric tumor- free mice when placed into a blastocyst that was then implanted into a pseudopregnant host [14]. Additional work by Mintz and Illmensee supported Brinster’s finding that cancer cells retain totipotency potential and respond to differentiation stimuli contributing to the development of “normal” somatic and germ-line tissues in tumor-free mosaic mice [10,12]. In 1976, Mintz and Illmensee transplanted single embroid bodies derived from OTT 6050 ascites teratoma cells into genetically marked blastocysts that were then implanted into pseudopregnant foster mothers. They were able to show the contribution of the single teratoma cells to the development of various tissues including germ layers using a variety of cell markers [10,12].

Interestingly, a specific correspondence between the microenvironment and the cancer cells is necessary for the malignant phenotype to be suppressed [18]. In 1984 Pierce and Wells showed that malignant transformation of cancer cells was suppressed only if the cancer cells were placed in contact with the trophectoderm cell layer within the blastocyst [18]. Furthermore, Papaioannou *et al.* in 1975 demonstrated that the blastocyst was able to suppress tumorigenesis only when the number of embryonal carcinoma cells was lower than 20 cells per injection [19,20]. Further studies by McCullough and collagues suggested that changes that disrupt a tumor non-permissive microenvironment structure and/or signaling such the ones that can occur with aging are important to suppress or promote tumorigenesis [5]. When the tumorigenic liver cells were injected in the liver of young rats, they were able to adapt and respond to the microenvironment stimuli and form normal hepatocytes. However, when the tumor cells were injected in the liver of old rats, cells formed fast growing, undifferentiated tumors similarly to when the tumor cells were injected in non-hepatic sites [5]. These findings suggest that there is a tightly regulated interaction between the tumor cells and the microenvironment and that tumor formation or suppression is dependent on the stability of this interaction. As previously suggested, a better understanding of the mechanism by which cancer cells can be “reversed” to a “normal phenotype” could be instrumental for developing treatments of cancer *in situ* or alternative to cytotoxic drugs [11,21].

Mammary epithelial cells that are incapable of growth can be rescued by the interaction with a competent niche and regenerate a functional mammary outgrowth *in vivo* [22,23,24]. Furthermore, it was demonstrated that the mammary niche was able to redirect mouse cells of non-mammary origins such as spermatogenic, neuronal and mesoderm-derived bone marrow cells to differentiate into mammary epithelial progeny and contribute to the development of mammary glandular regeneration *in vivo* [25,26,27].

To determine whether mouse cancer cells could be redirected to normal epithelial cell fate when mixed with a normal niche during the regeneration of the mammary gland *in vivo*, Booth *et al.* showed that Mouse Mammary Tumor Virus (MMTV)-neu-transformed cells mixed with wild-type mammary epithelial cells from primary mouse mammary epithelial cell cultures were redirected to participate in the development of a normal and functional mammary gland [28]. This result was extended to cancer cells of human origin, which were redirected from malignant to normal phenotype when mixed with mouse mammary epithelial cells in the context of an epithelium-divested mammary fat pad *in vivo*. Here, we discuss our studies where it was demonstrated that the normal mouse mammary niche is able to suppress tumorigenesis of human cancer cells of both embryonic (NTERA-2cl (NT2)) and somatic origin (*i.e.*, MDA-MB-231-GFP metastatic, MDA-MB-231BRMS-GFP metastasis suppressed, and MDA-MB-468 non-metastatic human breast cancer cells) and signaled the tumor cells to differentiate into human mammary epithelial cells, which contributed to the development of a functional mammary gland in both primary and secondary chimeric outgrowths [21,29]. Interestingly, our data demonstrate that human cancer cells are able to respond to the mouse normal tissue-specific developmental signals. We propose that cells in human cancers may be susceptible to control *in situ* by signals generated within developing mammalian tissues. 

## 2. Results and Discussion

### 2.1. Human Cancer Cells Contribute to the Regeneration of Tumor-free Chimera (Human and Mouse) Mammary Glands and Thus Respond to Normal Tissue-specific Developmental Signals

**Figure 1:**
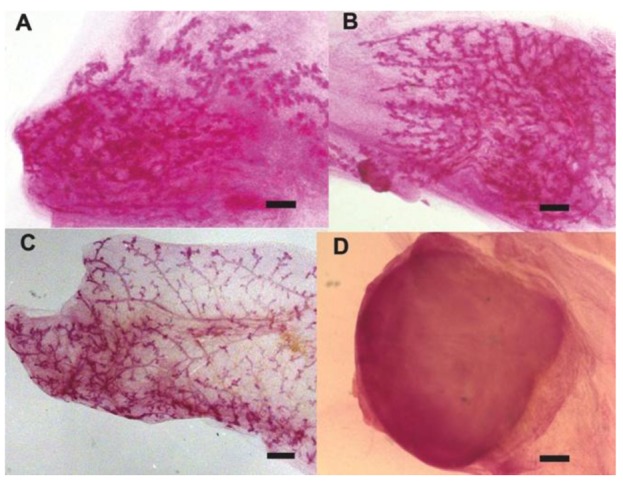
Tumor-free chimeric (human-mouse) mammary gland outgrowths**.** A-B) Representative whole mounted tumor-free mouse mammary outgrowths generated from the inoculation of both human embryonal carcinoma cells (NT2) and mammary epithelial cells. C) Representative whole mounted mouse mammary gland outgrowth generated from the inoculation of mammary epithelial cells alone (Control; 50K cells). D) Representative whole mounted mouse mammary fat pad of tumor generated by the inoculation of NT2 cells alone (10K). Mammary gland outgrowths were fixed in Carnoy’s fixative and stained with carmine alum. Scale bars = 2 mm.

Using the previously described technique of serial transplantation of mammary tissues into the epithelium deprived mammary fat pad of 3-week-old Nu/Nu mice, cells of non-mammary tissue origin were found to respond to the mammary tissue specific signals to form a normal and functional chimeric mammary gland [26,27,30,31]. Experiments where human cancer cells (pluripotent embryonal carcinoma (NT2) or differentiated breast cancer (*i.e.*, MDA-MB-231-GFP metastatic, MDA-MB-231BRMS-GFP metastasis suppressed, and MDA-MB-468 non-metastatic human breast cancer cells) where mixed in different ratios (1:5 or 1:50) with normal mouse mammary epithelial cells (MECs) derived from primary cultures and subsequently transplanted in the cleared fat pads of athymic nude mice gave evidence that human cancer cells interact with normal mammary epithelial cells and contribute their progeny in tumor-free chimeric mammary outgrowths which develop *in vivo* in mice (Figure 1A-B) [21,29]. The tumorigenic phenotype of the human cancer cells was confirmed in experiments done in parallel where tumor cells were transplanted at low density in the absence of mammary epithelial cells (Figure 1D) [21]. Fluorescence in situ hybridization (*FISH*) and immunostaining for human specific marker such as CD133 and keratin 14 (K14) identified human carcinoma NT2 cells integrated with mouse cells in the mammary epithelium within the chimeric outgrowths (Figure 3A-B) [21]. These findings provide evidence that human cancer cells are redirected from their malignant phenotype to differentiate into a “normal” mammary epithelial cell progeny during the regeneration of the mammary gland. 

The fact that neither the embryonal carcinoma NT2 or the human differentiated breast cancer cells (*i.e.*, MDA-MB-231-GFP, MDA-MB-231BRMS-GFP, and MDA-MB-468) developed tumors when transplanted in mixed ratio of 1:5 and 1:50 to normal MECs demonstrated that the mammary microenvironment is able to suppress the malignant phenotype of human cancer cells independently from their differentiation (embryonic or differentiated cultured cells) or tumorigenic stage (metastatic *versus* non metastatic breast cancer cells) [21,29]. Interestingly, the percentage of primary outgrowths derived from human breast cancer cells/MEC mixed ratio cell population (≥50%) was lower compared to the percentage of primary outgrowths derived from NT2/MEC mixed ratio cell population (>80%) [21,29]. It is possible that the differentiated state of the human cancer cells is the cause for the decreased efficiency of the primary outgrowths generation.

### 2.2. Human Cancer Cells Contain Multipotent and Self-renewing Progenitor Cells that Respond to the Mammary Niche Differentiation Signals

**Figure 2:**
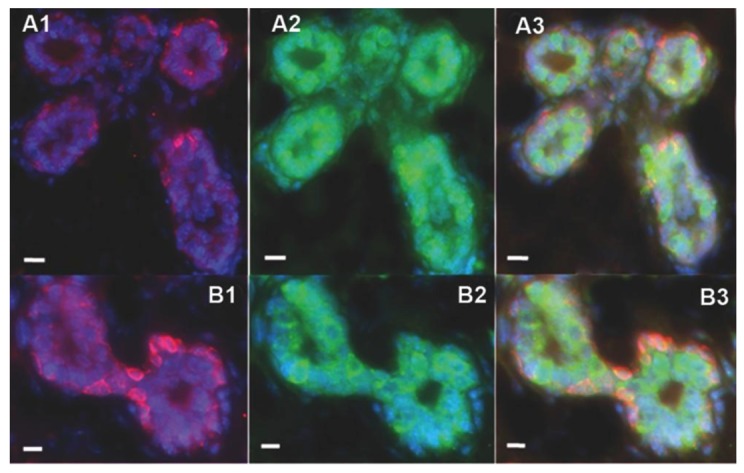
Human cancer cells are able to self-renew and contribute to both luminal (hK8-positive) and basal (hK5-positive) epithelial cell progeny in the chimeric mammary gland outgrowths. Immunofluorescence staining of primary (A1-A3) and secondary (B1-B3) mouse mammary outgrowths. Primary outgrowths were obtained by inoculating both human embryonal carcinoma (NT2) and normal mammary epithelial cells. Secondary outgrowths were generated by transplanting NT2/MEC primary mammary outgrowths fragments into epithelium-deprived fat pad of Nu/Nu mice. A1, B1) Human-specific myoepithelial markers keratin 5 (red). A2, B2) Human-specific luminal marker keratin 8 (green). A3, B3) Merge. All sections are counterstained with DAPI (blue). Scale bars = 10 μm.

Transplantation of mammary fragments in the cleared fat pad is a useful model to investigate the development of the mammary gland and the function and expansion of mammary stem and progenitor cells [31,32,33,34]. Immunostaining for the human specific luminal marker keratin 8 (K8) and the myoepithelial markers keratin 5 (K5) of the chimeric outgrowths demonstrated that human cancer cell contribute to both luminal and myoepithelial cells in the mammary outgrowths (Figure 2) [21]. These findings led to the hypothesis that human cancer cells contain ductal and alveolar progenitors that are able to respond to the differentiation stimuli of the regenerating mammary gland *in vivo* [32].

**Figure 3:**
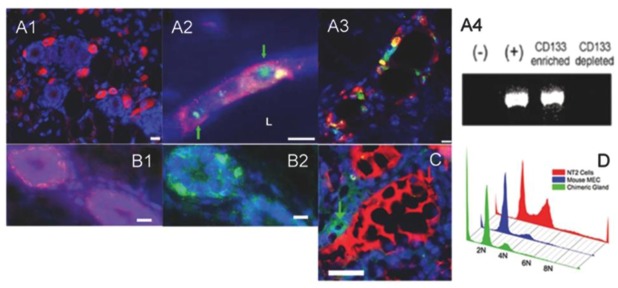
Human cancer cells identified in the tumor-free chimeric mammary outgrowths are hormonal responsive, secrete human milk proteins in the mammary ducts and do not show increased ploidy. (A1) Human-specific immunocytochemical staining for CD133 (red) shows CD133-positive NT2 cells present within the confines of the fat pad containing regenerated mammary ducts. (A2) Human-specific fluorescent *in situ* hybridization (green, nuclear; identified with green arrows) and human-specific immunocytochemical staining for CD133 (red) shows NT2 cells present within the mammary outgrowths. (A3) CD133-positive NT2 cells (red) differentiate into ER-alpha (green) luminal epithelial cells. (A4) PCR shows human specific Y-chromosome present in NT2 cells prior to transplantation (+) as well as in the CD133^enriched^ fraction obtained by magnetic sorting but not in normal mouse mammary epithelial cells (-) and the CD133^depleted^ fraction. (B1-B2) Basal cells express human K14 (red) and mouse K14 (green) in consecutive sections of the same chimeric duct. (C) Immunocytochemical staining of chimeric mammary outgrowth for human alpha lactalbumin (green) and mouse caseins (red). (D) Flow cytometry of propidium iodide stained cells demonstrate that chimeric glands (green) do not contain a greater proportion of cells with abnormal ploidy as compared to cultures of mouse mammary epithelial cells (blue) or NT2 (red). All sections are counterstained with DAPI (blue). Scale bars (A1) 20 μm, (A2) and (A3) 15 μm, (B1) and (B2) 10 μm, and (C) 25 mm.

Further characterization of the chimeric outgrowths identified CD133^+^ NT2 cells within the estrogen receptor alpha and progesterone receptor-positive luminal cells in mammary ducts (Figure 3A3) [21]. Similarly, immunostaining of the chimeric outgrowths for human mitochondria demonstrated human breast cancer cells localized in mammary ducts and lobules [29]. These findings were surprising since MDA-MB-231 and MDA-MB-468 cells lack expression of the estrogen receptor (ER) and progesterone receptor (PR) whose expression is required in the mammary epithelium for ductal elongation, side-branching and alveologenesis [35]. Presumably, the lack of nuclear receptor signaling of the human cancer cells is overcome by the signaling of the surrounding normal nuclear receptors expressing mammary epithelial cells. 

During pregnancy, the mammary gland goes through increased epithelial cell proliferation and alveolar differentiation in preparation for lactation [29,36]. The development of secretory alveoli comprised of luminal epithelial cells that produce and secrete milk proteins occurs in the intact mammary gland as well as in the implanted mammary outgrowths in impregnated hosts [29,36]. 

In order to determine if human cancer cell progeny formed secretory mammary epithelial cells, chimeric mammary outgrowths were removed following a full-term pregnancy at Day 2 of lactation. 

**Figure 4:**
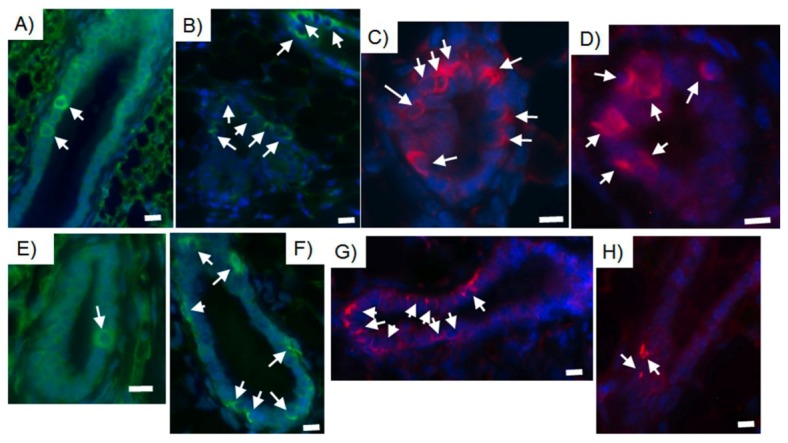
CD44 enriched as well as CD44 depleted human breast cancer cell population are able to integrate and contribute their progeny to chimeric mammary outgrowths. Immunocytochemical staining of chimeric mammary outgrowths obtained from the inoculation of either CD44 enriched or CD44 depleted MDA-MB-231-GFP breast cancer cells (10K) and mouse mammary epithelial cells (50K) A, E) Human keratin 8 (green); B, F) human keratin 14 (green); C, G) mouse keratin 14 (red); D, H) human mitochondria (red). A-D) CD44 enriched; E-H) CD44 depleted. Scale bars = 10 µm.

Outgrowths were found to completely fill the mammary fat pad and exhibited extensive development of secretory acini [29]. Immunofluorescence staining of chimeric mammary outgrowths tissues for human-specific milk protein such as alpha-lactalbumin demonstrated that human cancer cells contain alveolar progenitors that respond to the differentiation stimuli of the regenerating mammary gland during pregnancy and give rise to secretory alveolar cells (Figure 3C, Figure 4) [21,29,32]*.* As mentioned above, it was surprising that the MDA-MB-231 and MDA-MB-468 cells that lack expression of the estrogen receptor (ER), progesterone receptor (PR) were able to differentiate into luminal milk secretory cells in impregnated transplant hosts. Recent evidence indicates that expression of prolactin in the mammary epithelium itself is required for full lactogenic differentiation suggesting that the redirected human cancer cells can either synthesize human prolactin or respond to prolactin stimulation from the normal mouse mammary epithelial cells [37]. Immunostaining of secondary chimeric outgrowths in lactating hosts demonstrated that human cancer NT2 cells are able to synthesize prolactin [21].

Furthermore, human cancer cell progeny with a “normal” phenotype were identified not only in primary mammary outgrowths but also in secondary mammary outgrowths that were obtained by transplanting fragments of the primary outgrowths into the cleared fat pad of Nu/Nu mice (Figure 2B) [21]. Most importantly, no tumors were seen in either first or second generations transplants respectively. Interestingly, the number of human-derived cells significantly increased during the mammary gland regeneration. It was estimated a 60–660 fold increased in the number of human cancer NT2 cells in the secondary outgrowths compared to the primary outgrowths [21]. These data demonstrated that the human cancer cells are able to self-renew, integrate and contribute their progeny to the chimeric tumor-free mammary outgrowths during transplantations studies [29].

### 2.3. Human Cancer Cells and Host Mouse Epithelial Cells Do Not Fuse During Mammary Gland Regeneration

Cell-cell fusion is a biological event that can occur in somatic cells such as myoblasts and macrophages during tissue development and regeneration [38]. As discussed elsewhere, new evidence suggests that cancer cells can fuse with somatic cells during metastatic stages [39]. In tissue regeneration experiments with human multipotent cancer cells, human - mouse cell fusion can be a confounding factor and lead to an inaccurate interpretation of host tissue-specific differentiation of the implanted cells [22,40,41,42]. 

Evidence suggests that human-mouse cell fusion does not occur in our experiments [22]. First of all, the implanted cells are injected into the epithelium-deprived fat pad in absence of non-cellular matrix such as Matrigel or collagen [22]. The implanted cells are thus not held in close proximity *in vivo* [22,36,43,44]. Only a small percentage of the implanted cells will be able to aggregate and re-orient to form a nucleating growth cone that will then proliferate and expand radially to form the chimeric outgrowth [22,36,43,44]. Fluorescence *in situ* hybridization (FISH) for the presence of the Y chromosome of chimeric outgrowth tissue sections showed that male (NT2 cells) and female (mouse) cells existed side by side in mammary epithelial structures [21]. Similarly, microscopic examination of metaphase preparations isolated from second generation chimeric outgrowths developed from human breast cancer cells mixed the normal mouse mammary epithelium contained only human or mouse chromosomes in any individual metaphase but not both [29]. Furthermore, no aneuploidy or polyploidy was evident in any of the cellular preparations with NT2 cells isolated from the second-generation chimeric gland or from the co-cultured human NT2 cells and mouse mammary epithelial cells by fluorescence activated cell sorting of propidium iodide stained cells (Figure 3D) [21]. 

### 2.4. Tumor-initiating Cells Are Able to Obey to Normal Niche Signals

Although some tumors have been demonstrated to derive from clonal expansion, it is becoming clear that most tumors comprise heterogeneous cell populations that are at different stages of self-renewal, proliferation and differentiation potential [45,46,47,48]. Thus, it has been proposed that “time” and “location” of tumorigenic events within the hierarchy of cells will determine the expansion of some tumor cells over others [47,48]. Within the hierarchy of cells of the heterogeneous tumors, the so called “tumor-initiating cells” or “cancer stem cells” represent a rare and undifferentiated cell subpopulation and are thought to be responsible for tumor formation, progression, as well as tumor cell self-renewal [48,49]. Finally, recent studies have shown that there are similarities between the transcriptional profile of normal cells and colon cancer cells suggesting that colon cancers cells retain the potential of normal phenotype when placed in a permissive environment [45]. It was demonstrated that even xenografts tumors of single-cell clonal origin presented heterogeneity due to the differentiation of cancer cells into different cell lineages *in vivo* [45]. 

The results that human cancer cells of both embryonic or somatic origin were able to respond to the differentiating signals of the regenerative mammary niche independently from their differentiation state and metastatic potential, leads us to the hypothesis that tumor initiating cells retain the potential to be redirected towards a normal phenotype when placed in a permissive microenvironment such as the regenerative mammary gland. It has been shown that the breast cancer cells enriched for CD44^+^ expression have higher tumor promoting ability relative to CD44 depleted breast cancer cells possibly due the MAP kinase and PI3 kinase/AKT signaling cascades activation which subsequently increase cancer cell invasion, growth, motility, and survival [50,51]. Experiments where breast cancer cells were sorted for CD44 expression demonstrated that there was not a significant difference in the number of tumorspheres formed in low adherence conditions between the enriched and depleted CD44 cell populations [29]. However, it was observed that the depleted CD44 fraction showed a delay in tumorsphere formation and a decrease proliferation rate during the chimera outgrowths regeneration [29]. Most importantly, both CD44 enriched and CD44 depleted cancer cell populations were able to contribute to mouse/human chimeric mammary outgrowths (Figure 4) [29]. These findings indicate that either tumor-initiating breast cancer cells are more likely to be reprogrammed or alternatively that CD44 reprogrammed breast cancer cells are more likely to produce proliferative active, reprogrammed progeny [29]. 

One question that still remains unanswered is whether the normal niche terminally reprograms or just temporary redirects cancer cells to a normal phenotype. Experiments where erbB2 expressing cells were recovered by magnetic sorting from the secondary MMTV-neu chimeric outgrowths that were dissociated and cultured *in vitro* for six passages and transplanted in the cleared fat pad of Nu/Nu mice without normal mammary epithelial cells suggested that cancer cells retained their tumor -initiating ability in the absence of a normal regenerating niche [25]. It was observed that MMTV-neu cells retained the expression of erbB2 receptor in the chimeric outgrowths [25]. However, the receptor was not phosphorylated as seen in the erbB2 tumors [25].

Interestingly, experiments where cancer cells were mixed at higher density than normal MECs and subsequently transplanted in the cleared fat pads of athymic nude mice gave evidence that tumor suppression is dependent on the interaction of human cancer cells with normal mammary epithelial cells [28]. Presumably, a higher ratio of normal mammary epithelial cells to tumor cells is important to the formation of a niche that suppresses the activation of tumor cell signaling that drives tumorigenesis such as the phosphorylation of erbB2 receptor and promotes the development of normal mammary structure by compensating for the lack of nuclear receptors signaling of the tumor cells during the regeneration of the mammary gland *in vivo*.

Ongoing experiments are further investigating the mechanism through which the normal microenvironment suppresses tumorigenesis and if the tumor cells can be terminally reprogrammed to a normal phenotype. 

## 3. Conclusions

It was shown for the first time that human cancer cells either from an embryonic origin or a somatic origin can be redirected to a tumor-free phenotype and produce progeny capable of mammary epithelial cell functions upon the interaction with a developing tissue *in vivo*. The resulting interaction with the mouse mammary niche leads to the redirection of human cancer cell progeny to adopt a human mammary epithelial cell phenotype with an absence of tumorigenic activity. Interestingly, our findings provide direct evidence that the developmental cues in the mouse mammary gland and those present during human breast development may be interchangeable. 

It was demonstrated that human cancer cells give rise to a cell progeny that can differentiate into distinct mammary epithelial cell types, *i.e.*, luminal, myoepithelial, alveolar during chimeric mammary regeneration. Interestingly, even human cancer cells that lack of estrogen or progesterone receptor expression behave as hormonal responsive cells and secret human-specific milk proteins into the lumen of lactating hosts. We observed that human cancer cells proliferated independently in the formation of primary and secondary chimeras as shown by the absence of human/mouse cell fusion in the chimeric outgrowths as determined by FISH and propidium iodide. Overall our findings indicate that human cancer cells contain a subpopulation of cells that have multipotency and self-renewal potential independently from the tumor differentiation or metastatic stage and are responsive to signals from the mouse mammary microenvironment. 

Previously, evidence was provided for the existence of mammary epithelial stem/progenitor cells in the mouse mammary gland that are distinguished by their lobule-limited and duct-limited epithelial morphogenetic potential [23,36,52,53,54]. At present, there is no evidence for human breast stem/progenitor cells with similar capacities. Therefore, isolation and characterization of the human cancer cells present in the mouse/human mammary chimeras will be important in understanding the nature of the reprogrammed human cancer cell progeny and may lead to a new paradigm for control and treatment of cancer *in situ*. Currently, we are investigating which components present within normal mouse mammary tissues have the potential to redirect tumorigenic cells to normal cell function. Overall these findings suggest a significant amount of crosstalk between the tissue microenvironment and cancer cells and have identified a characteristic of human cancer cells that results in suppression of the malignant phenotype and in a response to “normal” developmental cues.
